# Small Bowel Perforation Secondary to Blister Pill Pack Ingestion: A Case Report

**DOI:** 10.7759/cureus.23895

**Published:** 2022-04-06

**Authors:** Ferdinand Rico, Alan Sbar, John Lung

**Affiliations:** 1 Department of Surgery, Division of Trauma, Acute Care Surgery, Surgical Critical Care and General Surgery, Mohawk Valley Health System - St. Elizabeth's Hospital, Utica, USA; 2 Department of Specialty Medicine, University of New England, College of Osteopathic Medicine, Biddeford, USA; 3 Department of Surgery, Texas Tech University Health Sciences Center, Amarillo, USA; 4 Department of Internal Medicine, University of Nevada, Reno School of Medicine, Reno, USA

**Keywords:** accidental ingestion, intestinal perforation, elderly people, blister pill pack, foreign body

## Abstract

We present a case of accidental ingestion of a foreign body-blister pill pack (FB-BPP) causing small bowel perforation in a patient taking aspirin and clopidogrel due to past history of coronary artery disease. A 71-year-old male presented in the emergency department (ED) with a two-day history of abdominal discomfort and loss of consciousness. His relevant home medication included aspirin and clopidogrel secondary to a history of coronary artery stents. Initial workup with emergent CT scan of abdomen/pelvis with intravenous contrast showed a loop of the terminal ileum with thickened wall and perforation. Incidentally, he was also found to have type II myocardial infarction. Emergent laparoscopic ileocecectomy with primary anastomosis was done. The postoperative course was unremarkable. The pathology report of the small bowel was consistent with a FB-BPP associated perforation.

FB-BPP ingestion with perforation is a rare occurrence. It occurs more often in the elderly with significant mortality. Our case of accidental ingestion of FB-BPP was confirmed retrospectively after histopathological evaluation, and complicated by type II myocardial infarction. Emergent laparoscopic bowel resection was done despite significant preoperative risks.

## Introduction

Accidental foreign body ingestion is common and can happen to any patient. However, foreign body obstructions have a higher risk in children [1], the elderly, prisoners, and psychiatric patients. Of the foreign bodies that are swallowed, most pass through uneventfully. Chicken bones, bone fragments, dentures, toothpicks, and cocktail sticks are among the most commonly ingested foreign objects [2]. Only 10-20% of cases require endoscopy for removal and only 1% require surgical intervention [3-5].

We present a case of accidental ingestion of a FB-BPP that results in a small bowel perforation. The case is complicated by the patient’s cardiac history and concern of a concurrent myocardial event. We describe this case with emergent surgical intervention, careful perioperative workup, close management of cardiac status, and prophylactic reversal of antiplatelet coagulopathy.

## Case presentation

A 71-year-old male presented to our ED as a transfer from an outside facility with a two-day history of abdominal discomfort and loss of consciousness. He appeared generally ill and in mild distress. Vital signs were essentially normal other than mild tachypnea with a respiratory rate of 24, although saturating well at 95% on room air.

**Figure 1:**
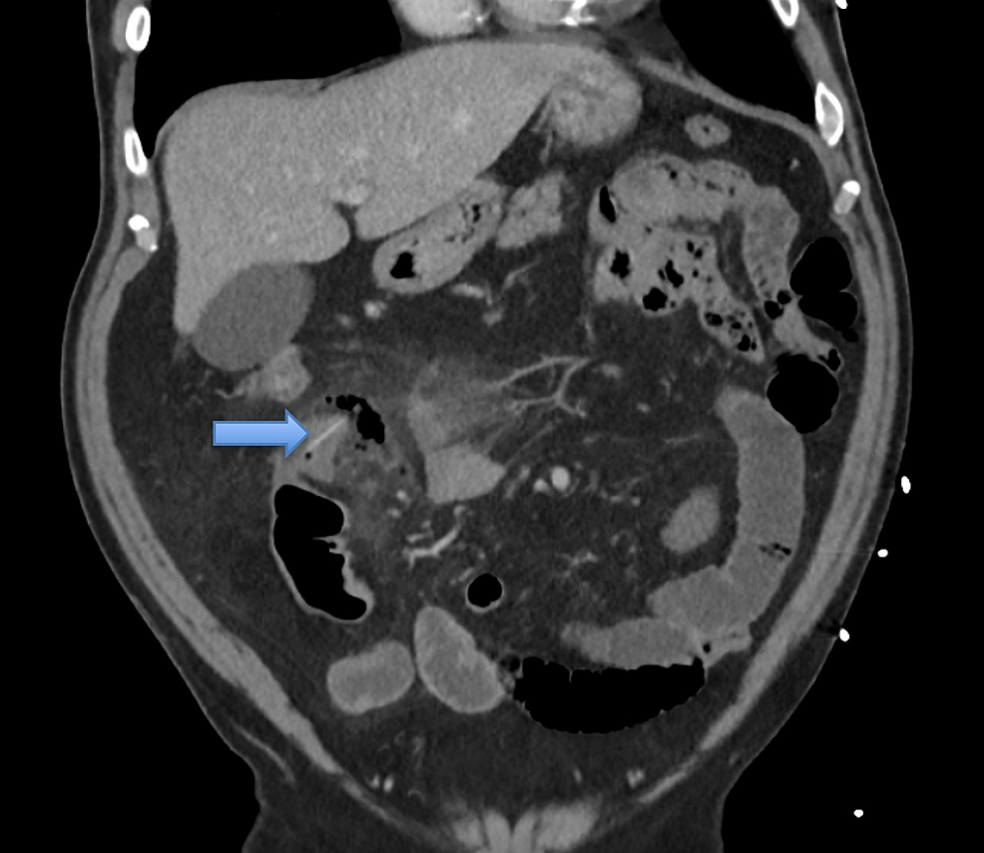
Coronal section of CT abdomen/pelvis with contrast showing perforation involving the terminal ileum. The blue arrow is pointing to the FB-BPP. The FB-BPP is not visible in the sagittal and the cross-section CT scan.

**Figure 2:**
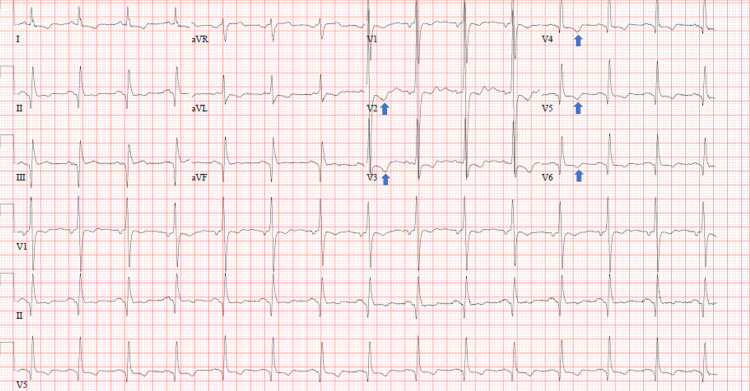
EKG showing T-wave inversions in leads V2 to V6 highlighted by blue arrows.

The patient reported two days of increasing right lower quadrant (RLQ) abdominal pain with subjective fever and chills. Concurrently, the patient complained of vague chest pain. He had a past medical history significant for hypertension, coronary artery disease, hyperlipidemia, epilepsy, three coronary stent placements, and a quadruple bypass. Significant home medications were aspirin and clopidogrel. His abdominal pain steadily worsened and he began having nausea and vomiting. The patient denied any current chest pain or diaphoresis during ED evaluation. An initial CT scan of the abdomen/pelvis with intravenous contrast showed perforated bowel involving a loop of ileum in the right lower quadrant of the abdomen with mural thickening and extraluminal gas. There was no foreign body described on the initial CT scan reading. In retrospect, further review of CT images showed a subtle foreign body consistent with blister pill pack perforations in the right lower quadrant site (Figure 1). Initial troponin was 5.86 ng/mL. An EKG showed T-wave inversions in leads V2-V6 consistent with anterolateral ischemia (Figure 2).

The patient was preoperatively managed on a nothing by mouth diet and crystalloid fluid maintenance. A nasogastric tube was placed on low suction. Intravenous broad-spectrum antibiotics were given. His home aspirin and clopidogrel were held and a heparin drip was started. An echocardiogram showed good left ventricular wall motion with an ejection fraction of 50-55%. A repeat troponin a few hours later decreased to 2.71 ng/mL. The elevated troponin was determined to be secondary to demand ischemia from an ongoing small bowel perforation. A left coronary angiography was deferred to prioritize surgical management of bowel perforation.

While the recommended management for localized small bowel perforation is surgical, operation in the face of a type II myocardial event posed a high morbidity and mortality risk. The cardiologist determined that since the patient’s condition was life-threatening, the patient could go to surgery without prior cardiac intervention.

**Figure 3:**
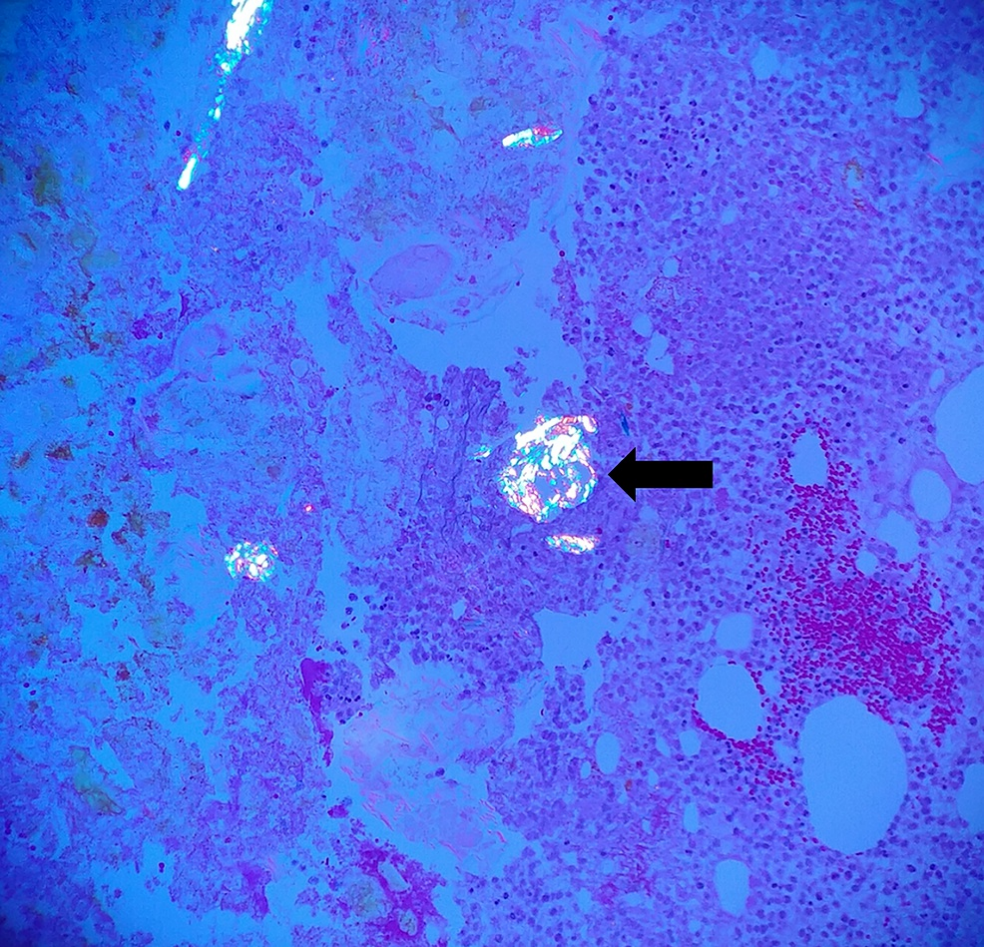
Polarized microscopy indicative of foreign material Multiple small amorphous portions of polarizable material shown as irregular blue-pink birefringent particles are scattered throughout the fibroinflammatory infiltrate. One area is highlighted by the black arrow.

**Figure 4:**
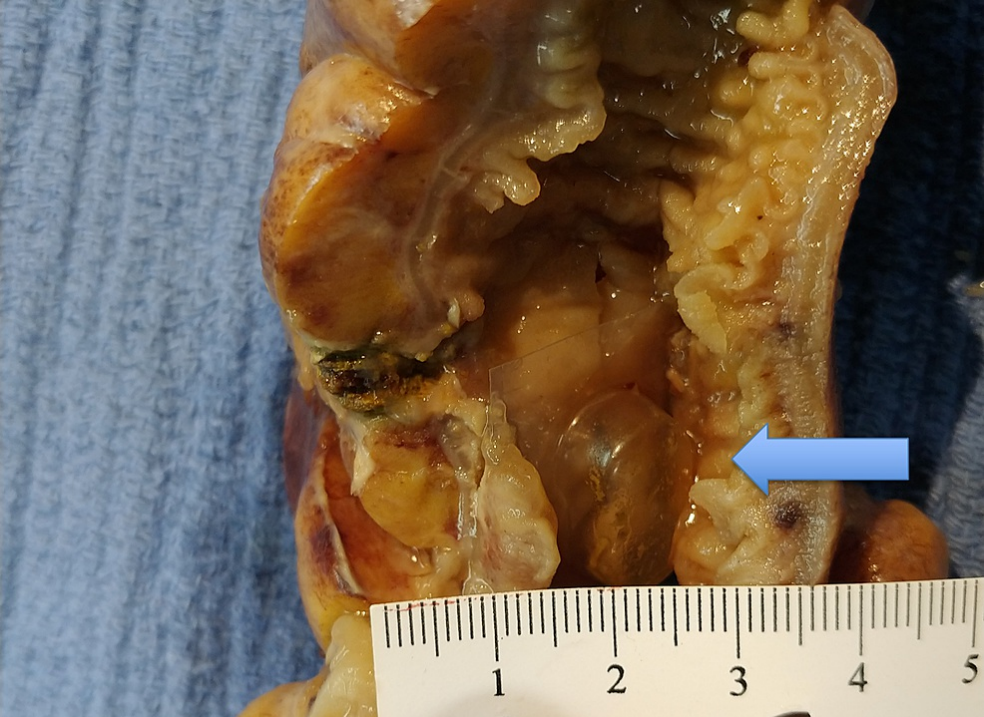
Perforated section of terminal ileum A transparent plastic blister pill pack is shown in the lumen by the blue arrow. The FB-BPP in the picture was overlaid to show its shape, plastic stiffness, and its four pointed ends. It also showed the size compared to the lumen of the terminal ileum.

The patient underwent a laparoscopic ileocecectomy with primary ileocolic anastomosis. Heparin drip was held two hours prior to operation. The patient received two units of platelets intraoperatively due to previous antiplatelet therapy. Histopathologic evaluation revealed a plastic foreign body lodged in the terminal ileum. Signs of acute on chronic inflammation and necrosis were consistent with foreign body-associated perforation (Figures 3-4). The polarized material in Figure 3 is indicative of foreign material, likely the pill itself.

After surgery, the patient was restarted on aspirin, a beta-blocker, and simvastatin. He was moved to the ICU for postoperative observation. The cardiologist planned no further intervention due to down-trending troponins and the bowel resection resolving the underlying cause of type II myocardial infarction. The patient’s postoperative course was uneventful. With the rapid return of bowel function, the diet was resumed in a few days. Clopidogrel was resumed on postoperative day 2 (POD). He was discharged home on POD four.

## Discussion

Foreign body impaction, obstruction, or perforation of the gastrointestinal tract most often occurs at areas of acute angulation or narrowing, especially at the level of the terminal ileum right before the ileocecal valve [6]. This is consistent with our patient’s bowel perforation occurring at the ileal anatomical narrowing as it approaches the ileocecal valve. There have been few reported incidences of this occurring with ingestion of FB-BPP [7-9]. Perforation of the gastrointestinal tract is a rare but extremely life-threatening condition. A delayed diagnosis portends a poorer prognosis [2]. When perforation of the bowel occurs, patients present with an acute abdomen. This includes peritoneal symptoms such as guarding, rebound tenderness, and abdominal rigidity [10]. CT scan imaging that may point to perforation includes localized inflammatory changes, extra-luminal free air, localized fluid collection or abscess, and a segment of moderately thickened bowel [2].

Our patient’s treatment was complicated by a type II myocardial infarction likely caused by the small bowel perforation leading to an oxygen perfusion mismatch. The prognosis of patients diagnosed with type II myocardial infarction is worse than that of patients diagnosed with a type I myocardial infarction (secondary to coronary artery disease), especially if the underlying demand mismatch is not corrected [11]. In most cases published in the literature, open surgery was performed for a foreign body perforation. In our case due to hemodynamic stability laparoscopy was used for the management of perforation. The risk and benefit ratio must be weighed heavily on FB-BPP retrieval, which can involve endoscopic surgery, minimally invasive laparoscopic surgery (our case), or open surgery depending on the surgeon’s experience.

Ingestion of FB-BPP with perforation is a rare occurrence. It occurs more often in the elderly and has a significant mortality rate even with surgical intervention. The elderly often have comorbidities including coronary artery disease, hypertension, diabetes, hyperlipidemia, and other chronic diseases that can complicate surgical intervention. This was true in our case as well. Ultimately, a CT scan revealed a small bowel perforation that required surgical intervention. The elevated troponin level, later determined to be caused by a type II myocardial infarction, delayed and complicated surgical management.

Blister pill packs are characteristically made of aluminum and plastic. They are usually hard with sharp edges and four pointed corners. They are not radio-opaque and thus may be missed in radiographic evaluation. Our case depicts careful perioperative multidisciplinary management. Whether urgent or emergent, minimally invasive surgery is preferable at the earliest possible time to mitigate morbidity and mortality. Perioperative management to optimize survival is of utmost importance in the geriatric population with multiple comorbidities. The geriatric population is likely to have dementia and be on polypharmacy. The use of antiplatelet therapy in the elderly further complicates surgical management, with no clear guidelines for reversal of antiplatelet therapies for emergent surgery. In our case platelets were given intraoperatively, though more literature is needed to guide platelet transfusions with patients taking antiplatelet agents [11].

## Conclusions

FB-BPP ingestion with perforation represents a poorly recognized but significant risk in the elderly. Most pill packs ingested pass harmlessly through the body. Management by FB-BPP retrieval, ranging from endoscopic to minimally invasive laparoscopic to open surgery, depends on the surgeon’s experience and the patient’s acuity. Our case demonstrates the benefit of laparoscopic interventions in the appropriate clinical context to minimize post-operative complications.
